# Viability of in-house datamarting approaches for population genetics analysis of SNP genotypes

**DOI:** 10.1186/1471-2105-10-S3-S5

**Published:** 2009-03-19

**Authors:** Jorge Amigo, Christopher Phillips, Antonio Salas, Ángel Carracedo

**Affiliations:** 1 Spanish National Genotyping Center (CeGen), Genomic Medicine Group, CIBERER, University of Santiago de Compostela, Galicia, Spain; 2 Forensic Genetics Unit, Institute of Legal Medicine, University of Santiago de Compostela, Galicia, Spain

## Abstract

**Background:**

Databases containing very large amounts of SNP (Single Nucleotide Polymorphism) data are now freely available for researchers interested in medical and/or population genetics applications. While many of these SNP repositories have implemented data retrieval tools for general-purpose mining, these alone cannot cover the broad spectrum of needs of most medical and population genetics studies.

**Results:**

To address this limitation, we have built in-house customized data marts from the raw data provided by the largest public databases. In particular, for population genetics analysis based on genotypes we have built a set of data processing scripts that deal with raw data coming from the major SNP variation databases (e.g. HapMap, Perlegen), stripping them into single genotypes and then grouping them into populations, then merged with additional complementary descriptive information extracted from dbSNP. This allows not only in-house standardization and normalization of the genotyping data retrieved from different repositories, but also the calculation of statistical indices from simple allele frequency estimates to more elaborate genetic differentiation tests within populations, together with the ability to combine population samples from different databases.

**Conclusion:**

The present study demonstrates the viability of implementing scripts for handling extensive datasets of SNP genotypes with low computational costs, dealing with certain complex issues that arise from the divergent nature and configuration of the most popular SNP repositories. The information contained in these databases can also be enriched with additional information obtained from other complementary databases, in order to build a dedicated data mart. Updating the data structure is straightforward, as well as permitting easy implementation of new external data and the computation of supplementary statistical indices of interest.

## Background

Many areas of study in genetics, such as human population genetics, are based on genomic diversity, and this variability can only be measured reliably by studying large amounts of data. These studies are only realistically available to big organizations and institutions, and their resulting databases become important data resources for many other genetics projects. Therefore the ability of individual researchers to browse large databases such as HapMap  or CEPH  is critical meaning any improvement in data management can be as valuable as the data itself.

The availability of different repositories of human variation represents an aid for researchers on one hand, but an inherent obstacle to their thoughtful combination on the other. Merging data from different databases, even if very similar, represents a major challenge for most users. An important aspect of online data obtained for population genetics studies is that not all databases reference the same material, with each database accessing different populations with their own samples and sample size, so often populations with the same description must be treated separately.

### Data marts

A common trend in the field of data repositories is the adoption of data marts, comprising specialized subsets of entire databases designed specifically to answer focused questions [1]. Data marts benefit from a streamlining of the dataset, which avoids querying more data than is needed. This exploits the data stored in a repository, but can use unique structures or summary statistics generated specifically for an area of research. Thus, data marts benefit from the existence of a broadly based database, are less general than a repository, but provide more effective and efficient support for tailored uses of the data.

The use of these data structures is indicated in enterprise-wide data, when operated by departments whose database structures are subject to occasional modifications [2]. The same idea can be ported to any database structure, since it can integrate and consolidate all relevant data into a single data mart without high operational overheads.

Our implementation consists of a large-scale rewriting of all the databases of interest in which we prepare the data to be queried for population genetics purposes, standardizing and normalizing their formats into a common and simplified structure while enriching the data mart with complementary information.

### Large genotyping databases

With the current availability and quality of online genome databases it is increasingly feasible to conduct population genetics research using *in-silico *resources [3] as an adjunct to the traditional strategy of sampling populations of interest and genotyping a range of polymorphic markers. Population genetics studies are not co-incidental to the characterization of the human genome or analysis of complex disease but are critical in informing how such analyses should be properly framed with reference to the level of susceptibility, the particular allele frequency distributions and the demographic history shown by a population. Autosomal SNPs, while individually less informative *per se *in population variability terms than e.g. mitochondrial and Y-chromosome loci or autosomal microsatellites, benefit from being densely distributed and well characterized at the sequence and functional level. The characterization of the population variability of SNPs is now catching up with information about their genomic role or their ability to provide landmarks for association studies, promoted in large part by detected differences in linkage disequilibrium patterns between population groups or in admixed populations [4,5]. The evolution of HapMap has illustrated the increased emphasis on extending large-scale genomic projects towards a broader scope of populations studied rather than loci genotyped. HapMap Phase III has almost tripled the study populations from four to eleven while the SNPs studied have been consolidated more than expanded.

### Text parsing

The parsing of large amounts of data has been a core approach in bioinformatics from the very beginning. In fact, programming and scripting languages with optimized pattern matching capabilities have been available for a long time (notable examples include Perl and Python), and the use of their built-in regular expressions makes it easier to deal with large numbers of extensive plain text files [6,7]. Current text-mining approaches benefit from these algorithms, which are flexible yet powerful.

All the main public genetics databases provide compressed-format dumps of their data for in-house processing, so once the raw data of interest is available as text files it only requires some familiarity with their format to inspect the required fields from each respective data dump. Although the amount of information to be processed does not generally represents a limitation as the parsing process will be completely automated, efficient programming allows best use of computer resources.

## Methods

By building a data mart for population genetics we aimed to improve population data management regardless of size, while consolidating data from different sources by including a number of complex, pre-calculated fields, data structures, and function libraries [8]. Our main goal is to provide a flexible and reliable single repository where the major databases of this field of study can be represented, to form the basis for creating custom queries both within and between each database.

### Population based data resources

**Figure 1:**
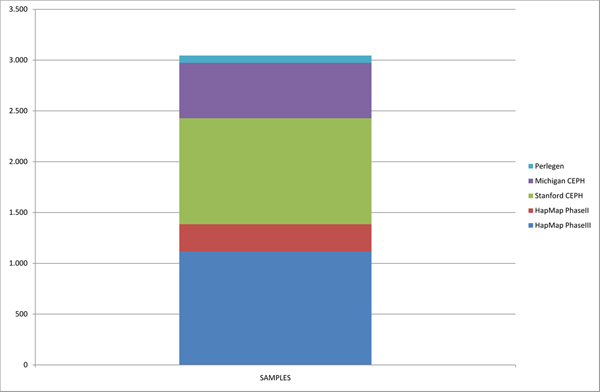
**Number of samples present on the data mart**. There are 3045 samples represented on our repository. The distribution of the number of samples per database vary from the most ambitious ones such as HapMap Phase III and the Stanford HGDP that contain over 1000 samples each, to others with less variation representation such as Perlegen, with only 71 samples on it.

**Figure 2:**
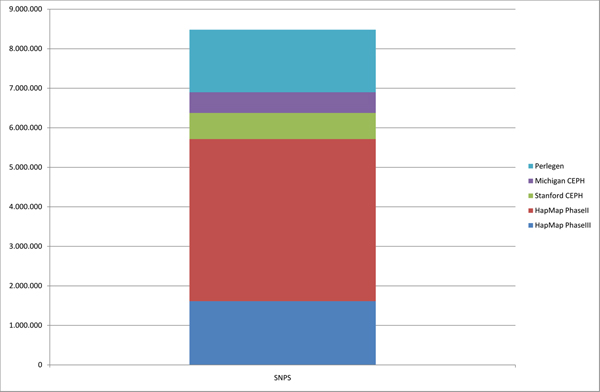
**Number of SNPs present on the data mart**. Around 8.5 × 10^6 ^SNPs are processed from the different databases, although these SNPs are not independent. Considering the SNP codes sharing presented on Table 1, where the HapMap Phase II database is the major SNP contributor, the number of distinct SNPs represented on the data mart is close to 4.5 × 10^6^.

**Figure 3:**
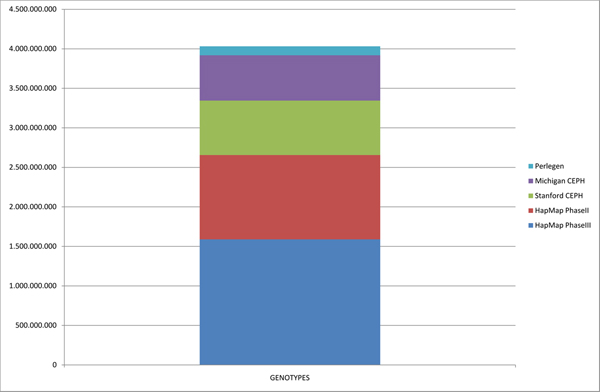
**Number of genotypes present on the data mart**. A total of above 4 × 10^9 ^genotypes are summarized on our data mart. Although the number of samples on Perlegen is not very high, its SNP coverage is, transforming this database along with both HapMap phases into the major genotyping contributors with over 10^9 ^genotypes each.

Many online databases cataloguing human variability provide population information about the samples studied, notably HapMap [9,10], Perlegen [11] and the CEPH foundation [12]. They also provide the raw data that underlines each online database for downloading and local analysis. We have chosen the raw data from the above repositories to be included in our data mart: the stable Hapmap Phase II release 24 and the preliminary released Phase III version, the Stanford and Michigan University CEPH-HGDP (Human Genetic Diversity Panel) SNP genotyping data (although the two datasets are significantly overlapping in SNPs and samples [13]), and the Perlegen dataset. Figures 1, 2 and 3 outline the genotype data of each database, showing the overall amount of data to be managed when building a query.

The datamart created is supplemented with dbSNP [14] data to map all the above databases to the same common reference. This overcomes issues of databases being mapped to different dbSNP builds, and automatically prepares the mart for incorporated any future SNP databases. Table 1 shows the overall number of SNP codes shared among all the processed databases.

**Table 1:** Shared SNPs among the different databases.

	**dbSNP**	**HapMap II**	**HapMap III**	**Perlegen**	**Stanford**
HapMap II	4097825				
HapMap III	1611772	1549224			
Perlegen	1585334	1267374	682386		
Stanford	660823	660060	658947	294956	
Michigan	525859	525307	525011	242910	525909

The number of common SNP codes has been taken from direct inspection of the raw data, after being mapped to the same reference SNP code by merging the dbSNP information. The numbers shown are the SNP codes that match among all the databases processed: dbSNP build 129, HapMap Phase II and III, Perlegen, and the human genome diversity panels from the universities of Stanford and Michigan.

### Data format analysis

Scrutiny of the publicly available core population-based SNP databases indicates similarities that all share: they are all dumped in plain text arranged by columns, and these columns are divided into descriptive data plus the genotypes themselves. We can use the descriptive information to include as much detail in the data mart as required, but the main aim of processing these files is to read them at the genotype level and to store genotyping calculations into appropriate variables.

### Hapmap, Stanford CEPH and Perlegen: tabulated format

Hapmap, Stanford and Perlegen use a similar format for their raw data, comprising genotyped individuals samples *versus *SNPs table, and they only differ in the character used to separate the columns (HapMap Phase II, III and Perlegen use blanks, Stanford tabulations) plus the amount of descriptive columns to characterize each SNP line. Once the amount of descriptive columns is stated, it is possible to jump to the first column of genotypes and read them in full. The format used by the Stanford CEPH comprises samples *versus *SNPs table, without additional information. In contrast, Perlegen provides some extra columns such as chromosome position or available alleles, but does not refer directly to reference SNP codes but to internal ones requiring an auxiliary translation file. Finally, HapMap goes further by providing Perlegen's additional data with additional columns such as strand information or the genotyping protocol used for single SNPs.

### Michigan CEPH: structure format

The reference data from the Michigan University is formatted following the requirements of the population stratification analysis software Structure [15], which comprises several header lines containing the SNP list amongst other information, then pairs of lines for each sample containing the first and second allele per SNP in the first and second line of the pair respectively. Parsing this database is therefore completely different from the rest. Once this SNPs *versus *samples data structure can be processed, along with the converse samples *versus *SNPs structure, any upcoming database of genotypes will presumably only require a slight adaptation of either structure reading module, making this system very flexible in terms of data mart expansion.

### Design of scripting variables

The biggest challenge of the parsing script design is to allow the data structures to be as versatile as needed but consuming as little computational resources as possible; specifically, in terms of processor running time and memory required. Once the genotypes are highlighted in each file format, the script should store as much relevant information from them as possible for extensive later use. For this reason, it is important to reduce to the minimum the indexing level of the hashes used in the script making them fast to build up and query while minimizing demands on memory.

The data itself is already contained in the raw compressed data files, so the proposed data mart will only contain metadata extracted and calculated from them, such as summarizing counts and percentages. For this reason, all the counts in the script are internally structured in hashes, which are indexed by population and re-used for each chromosome. In this way the script optimizes the memory consumption, and at the same time allows structuring of results into populations. By storing this metadata, which can be as extensive as desired, we have constructed a very detailed data mart queried independently of the original data and fully focused on our field of interest.

## Results and discussion

All processed data is placed into a MySQL database to contain all statistical results simply indexed by SNP code. The main challenge of the data mart design is the formation of a global design that allows the combination of SNP resources with different stuctures. It involves processing large SNP databases that require an efficient data indexing to minimize access times and memory requirements while retaining the versatility of the created scripts for new databases.

Although some databases may contain extensive additional information about SNP loci, it is worth noting that we focused on genotypes alone so the data files indicated on Table 2 represent the minimum number of files needed to build the population data mart described. Therefore files contain raw genotypes, SNP code translations (Perlegen data dumps contain internal codes only) or information about the samples familial relationships with others in the same set, required when building independent statistics.

**Table 2:** Raw data resources needed for the data mart creation.

**DATABASE**	**RESOURCE**
dbSNP	reference alleles from b129_SNPContigLoc_36_3.bcp.gz at
	ancestral alleles from SNPAncestralAllele.bcp.gz and Allele.bcp.gz at and
	chromosome positions, validation status and loci from reports at
	merged snps from RsMergeArch.bcp.gz at
HapMap II	
HapMap III	
Perlegen	
Stanford	
Michigan	

Processing each database requires its own raw data. The following are all the file sets, indicated by database, that were processed in order to build the described data mart. All of them are publicly available online.

### dbSNP, as a reference database

In the first instance mapping information is taken from dbSNP to form the reference template for other databases. The data for each SNP is obtained by parsing the files described in Table 2 to generate a list of SNPs per chromosome with descriptive information from dbSNP, such as the ancestral allele, to characterize each locus.

Processing the dbSNP database is perforrmed once per build and takes ~8 hours on a standard computer. The data is then merged with the SNP list of population databases included in the data mart, taking 10 to 15 minutes per database. This process is run when a population database or dbSNP is updated.

### Unifying chromosome mapping and SNP codes

There are two main problems when trying to compare the same SNP information from different databases: firstly, although a SNP may be named equally in multiple repositories its chromosome location may not coincide due to mapping changes between dbSNP versions; secondlt the SNP may just be named differently. The first issue will only affect queries by location, but it can be easily solved by always using the chromosome location from a chosen dbSNP build, not necessarily the last one, as consistency is the only requirement. However use of different SNP codes to refer to the same locus requires translating them into a common reference, either because of using internal SNP codes as Perlegen does, or because of being mapped to an older dbSNP build not reflecting the latest SNP label merges or renames.

The logical way to solve both problems is to map all the databases to the most recent dbSNP build. This will not only permit multiple chromosome positions, but also allows the data mart to contain updated SNP codes. By parsing the locations from the chromosome reports of the last dbSNP build and merging information from previous builds, we generate a mapping reference to use with the SNP lists from each processed database ready for placing into the data mart.

### The oriented reference allele

Although the major issues for SNP comparison are addressed, we also wanted to include a system to unify the strand interogated by the reported genotyping assay,. Although the strand information was part of the dbSNP raw data, a proportion of SNPs in repositories were genotyped on the complementary strand and required a mapping reference for allele calls. Therefore we opted to use the reference allele. The reference allele is arbitrary when working with genotypes, but it is still used to sort the genotyping alleles. So from the reference allele the direction is discerned and adjusted appropriately in each database. This orientation reference can be used to adjust the reporting of alleles from different databases that detect opposite strands.

### Data mart creation and structure

The set of scripts designed in the present study is able to process the major SNP databases and to generate a normalized data mart for them all, using relatively few resources. The most critical script processes the raw data from each database, as it has to be powerful but flexible. The script must read databases in the given format and calculate several statistical indices.

There are two main reading modules to handle samples *versus *SNPs or SNPs *versus *samples formats, and generate data uniform data structure. The statistical module follows and creates all the statistical summaries, from the simplest allele frequency estimates to more complex metrics of population differentiation, by building simple internal counts and summarizing them at the end. Finally, a writing module is in charge of generating a CSV file per population plus a list of the SNPs and the populations processed.

Once all the summarized data is written on these CSV files, a small script merges the SNP lists of each database with the additional SNP descriptive data from dbSNP. The merging script generates extra CSV files if relevant, such as the SNP codes merged or SNPs removed after comparison to dbSNP. The CSV files are loaded into a MySQL database by another script that generates the SQL commands to create each table definition, with SNP codes indexed to speed up any later inspection.

### Maintaining the data mart

The frequency of updates of the databases currently accessed is very low while dbSNP updates annually. HapMap data is rebuilt twice a year in contrast to Perlegen and the two CEPH databases, which appear to be static. Therefore new HapMap releases invlove running our complete pipeline (~2 hours on a standard computer), but a new dbSNP release requires only the merging script on each database SNP list, and updating only the SNP tables of the data mart (~1 hour for all the databases present).

**Figure 4:**
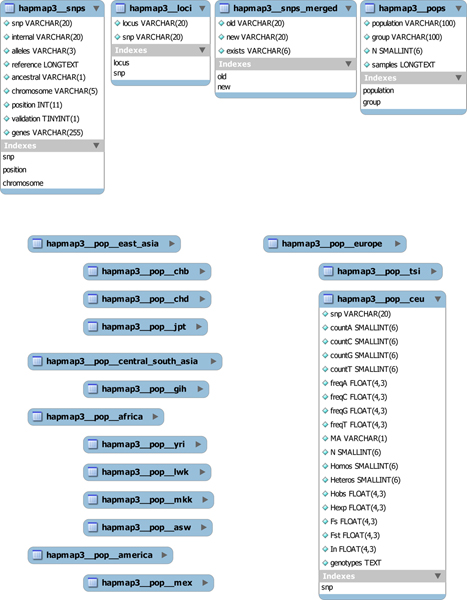
**Data mart tables for the HapMap Phase III database**. Each database summarized is present on the data mart as a set of tables containing descriptive SNP information and population specific calculations. Every database will have all the table structures expanded at the top of the image, and the amount of the population specific ones shown with the "__pop__" label will depend on the amount of populations covered by the database. Only the CEU population table structure has been expanded on the image, but the rest of the population tables share the same structure that allows filling each population SNP with all the available counts and calculations performed by the raw data processing script.

The interdependency of each database is outlined in Figure 4, where only the HapMap Phase III substructure of the data mart is shown. Each database replicates this structure, illustrating how compartmentalized the data mart is. Therefore it would be easy to add a new SNP database or to update existing ones.

We have implemented and summarized the most common population statistical indices. If new statistical indices are required the script processing the raw data needs to be updated, the statistical module would require modification, and the whole set of databases re-processed to reflect these changes. This represents a major update effort, as the entire data mart has to be rewritten, but in fact only requires a day of processing due to the flexibility the processing pipeline developed.

### Consumption of resources

**Figure 5:**
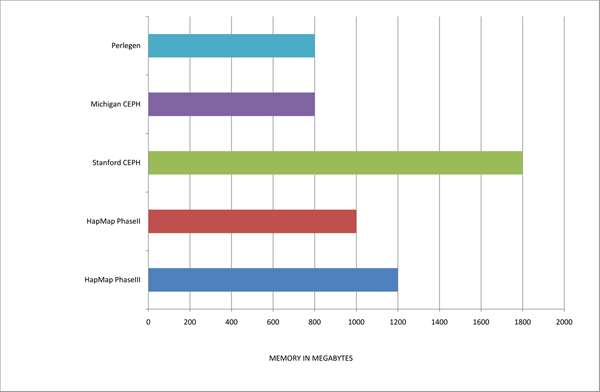
**Memory needed to process each database**. The memory required to deal with different databases depends not only on their number of samples and SNPs, but also on the raw data files structure. Although not more than 1 GB of memory has been enough for most the databases, the Stanford data needed some more due to its high population coverage. The fact of containing so many samples and representing so many populations on single files per chromosome forced the processing script to store plenty of indexed information that demanded high computational resources. The optimized design of the variables, along with the strict memory handling of the script, minimized this issue never requiring more than 2 GB.

**Figure 6:**
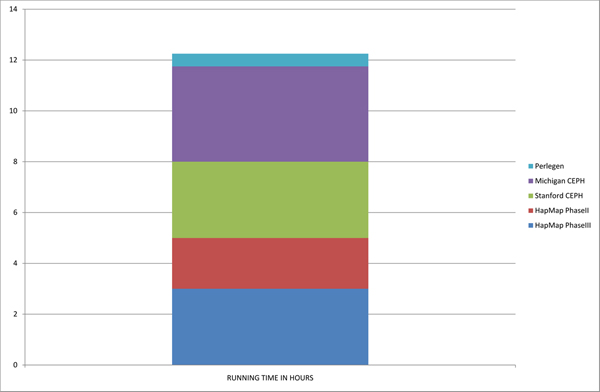
**Databases' processing times**. Cumulative time is presented, taking 12 hours to deal with all the available databases, although each task is independent from the others and therefore can be run in parallel. The maximum time would then be the 4 hours that the Michigan data needs to be processed.

One of the main aims of this project was to develop a tool for extracting the most relevant data from large SNP databases in such a way that a non-expert user can successfully complete the task using a standard computer. Firstly we focused on the memory requirements so the variables structure was designed to be as simple as possible, and secondly we optimized the main internal loops present in the script enabling the running time to be reduced to a minimum. This optimization led to the results displayed in Figures 5 and 6, indicating that all five major reference databases are processed in just 12 hours in total on a standard computer (although these are completely independent tasks), and that the maximum amount of free RAM needed for the computer is 1.8 GB (due mainly to the combination of a large number of samples and populations in the Stanford and Michigan CEPH data). Without considering the data that has to be extracted from dbSNP to be used as the mapping reference, the total number of genotypes currently contained in the data mart is above 4 × 10^9^. The total disk space needed is 16 GB, which is relatively small considering the size of the databases contained, and that half of that size is dedicated to the storing of the raw genotypes retained for user downloads.

### Posterior data mart use

The creation of a specific and smaller repository from larger ones was motivated by the need to avoid processing irrelevant data present in many repositories, as well as fully controlling its format and structure. We relied on text mining approaches when processing large variation repositories in order to obtain all available genotyping data for each SNP, and then summarizing that information to store it in a lean yet flexible data mart.

As an illustration of the marts use, a researcher might want to study the admixture of European and African populations in SNP rs2789823, amongst others, by querying all the variation repositories available. Normally this would mean browsing each database in turn while adapting to their different interfaces and data formats, and annotating the relevant information. Our tool alternatively mines available information for the SNP, and pre-calculates the relevant statistical indices that allow interpretation of the SNP variability. Therefore only the populations need to be selected. In the example given, our datamart rapidly creates output that indicates the Perlegen African American population at rs2789823 has a high degree of European admixture when compared with the HapMap African population (Yoruba of Ibadan, Nigeria).

Once the data has been summarized and organized, the next logical step is to build custom tools to query the new data structure and generate statistical metrics. The web-based tool SPSmart [16] has been designed with the aim of exploiting the previously generated data. It is therefore an online interface for the data mart built from the previously described reference databases, and is mainly focused to meet the routine analysis demands of population geneticists. These include comparing populations from different databases, inspecting allele frequencies across current available population databases, or studying the genetic differentiation amongst various combinations of populations.

### Future work

Since processing each database is completely independent from the rest, we can distribute the work through a parallel computer or through a grid system. Due to the large size of the raw data to be processed, currently around 2 GB of compressed text files, we have chosen the first option in order to minimize the latencies that data transfers through the network may cause. We are currently implementing our pipeline on a shared memory node system with SMP NUMA architecture available at the Supercomputing Centre of Galicia (CESGA; ). We can take advantage of the fact that the CESGA also hosts our data mart and the networking among the different machines is optimal. Our first tests show that this type of implementation is fully reliable, as we are obtaining similar benchmarking results compared to local runs, and our goal is to build a static pipeline structure on this supercomputer that would not only dramatically reduce our dependency on the network for large data uploads when updating any database, but also have a dedicated machine for our needs.

We have designed the data mart for handling high-throughput SNP genotyping data in such way that allows easy expansion, not only in terms of the databases accessed, but also in terms of new statistical indices that will be of interest to researchers. Thus, new repositories can be added to the data mart structure simply by adapting the reading module, while implementation of new statistics can easily be accomplished by adding the necessary formulae to the data and writing module of the processing script.

## Conclusion

There is a wide range of autosomal SNP genotypes resources freely available in public databases, each presenting their own storage procedures and formats. Due to this lack of homogeneity it is difficult to adapt to each database interface requirements and, with the software currently available, it is impossible to combine such disparate results for meta-analysis. Here we have shown that it is viable and highly efficient to work directly with the raw data of each repository to build data mart tailored to population genetics needs that uses in-house computational resources.

Adapting these major variation repositories in such a lean and versatile manner is a novel and ambitious approach to SNP based population genetics analysis, as it deals with a vast amount of information but is able to generate a flexible resource to obtain population statistics of any population or custom population group. Once the raw data is pre-processed, it is relatively easy to compute new statistical indices of interest and where new inter-population comparisons can be made. In addition, the strategy presented here allows the direct combination of different SNP genotyping repositories in a straightforward manner.

## Availability and requirements

• Project name: SPSmart

• Project home page: 

• Operating system: Platform independent.

• Programming languages: Perl and SQL.

• Type of access: all the scripts provided to generate the described data mart are freely available for non-commercial use.

## Competing interests

The authors declare that they have no competing interests.

## Authors' contributions

JA carried out the design and implementation of the described data mart, as well as the programming of the text parsing engine, and drafted the manuscript. AS, CP and AC participated in the design of the software and the database selection, and helped to draft the manuscript. All authors read and approved the final manuscript.
